# African leaf (*Vernonia amygdalina*) extracts improve Japanese quail (*Coturnix coturnix japonica*) carcass traits

**DOI:** 10.14202/vetworld.2023.773-778

**Published:** 2023-04-15

**Authors:** Sri Kismiati, Teysar Adi Sarjana, Luthfi Djauhari Mahfudz, Dwi Sunarti Prayitno

**Affiliations:** Department of Animal Science, Faculty of Animal and Agricultural Sciences, Diponegoro University, Tembalang Campus, Semarang, Central Java, Indonesia

**Keywords:** chemical and physical, cholesterol, fat and meat color, moisture, non-carcass percentage, protein

## Abstract

**Background and Aim:**

The *Vernonia amygdalina* Del. leaf extract (VALE) contains several natural antioxidants, including flavonoids, which effectively ameliorate cholesterol levels while improving quail carcass traits and meat quality. This study aimed to evaluate the effects of VALE on Japanese quail (*Coturnix coturnix japonica*) carcass traits and meat.

**Materials and Methods:**

In total, 260 Japanese quails (aged 5 weeks and body weight = 129.1 ± 2.2 g) were raised in an open-sided house and randomized to four VALE treatments: T0: Control, T1: 10 mL/L, T2: 20 mL/L, and T3: 10 mL/L in drinking water. After 12 weeks, carcass traits and chemical and physical meat qualities were evaluated.

**Results:**

*Vernonia amygdalina* leaf extract in drinking water exerted significant effects (p < 0.05) on carcass weight, cholesterol levels, and meat water holding capacity (WHC) without significantly affecting carcass and non-carcass percentages, moisture, protein, fat, and meat color qualities. The highest carcass weights and lowest cholesterol levels were identified in the T2 group, while WHC improved in the T3.

**Conclusion:**

Thus, VALE supplementation (20 mL/L) to quails improved carcass traits, especially cholesterol levels and carcass weights.

## Introduction

Quail is a domesticated bird of important economic value. Its meat and eggs have specific flavors, with Japanese quails (*Coturnix coturnix japonica*) producing one of the best meats among domesticated quails. The average adult weight is approximately 200 g, with birds easily stressed due to environmental conditions. Quail meat contains nutrients which provide valuable sources of animal protein. In Indonesia, the quail agri-business is unique, in some case female quail of laying type are harvested even during egg production phases. Thus, apart from egg production, carcass production traits and meat quality have become serious issues during the rearing processes. Studies have indicated that 8-week-old Japanese quail meat contains high protein and fat levels (23.32–24.55 g/100 g and 1.1–1.30 g/100 g, respectively) [1], but high cholesterol levels (71.07–87.0 mg/100 g) [1, 2]. However, quail farming in Indonesia faces highly fluctuating temperature and humidity conditions; too high or low temperatures affect bird health, growth, product quality [3]; carcass quality [4, 5]; body weight, carcass weight, carcass percentages [6, 7]; immunity, nutrient digestibility, and product quality [8, 9]. Environmental farming factors cannot be ignored and have key roles in determining performance and meat quality, especially when oxidative stress mechanisms and potential negative impacts are considered. Long-term bird stress also disturbs protein synthesis in different muscle groups and reduces protein breakdown and disposition in tissue [5]. To cope with stress effects, providing plant-based feed additives is a safe and viable solution [8]; using natural ingredients in Japanese quail diets improves carcass quality characteristics and meat quality [10].

*Vernonia amygdalina* Del. leaf extracts (VALE) contain phenolic acid and flavonoid compounds [11]. Phenolic acid is an immunomodulator, anti-mutagenic, and anti-inflammatory agent [12]. *Vernonia amygdalina* is a natural source of antioxidants, including phenols, flavonoids, anthocyanin, proanthocyanin, and tannins [13], which combat oxidative stress [14]. Adding flavonoids to broiler diets increased villus height and ratios, duodenum and jejunum crypt depth, growth, and carcass composition [15]. Natural antioxidant supplementation to quail feed reduced heat stress, significantly increased body and carcass weight [16], and increased meal quality [17]. In broilers, Saracila *et al*. [18] used antioxidants to reduce stress effects and successfully increase protein content. Japhet and Godgift [19] reported that VALE supplementation (50 mL/L) to drinking water increased broiler carcass performancebut did not increase the laying hens pullet performance. Supplemented *V. amygdalina* (30 mL/L) juice in drinking water increased carcass weight but percentages were not significantly different [20]. Suhaemi and Hidayati [21] showed that African leaves reduced cholesterol levels in broiler meat. Efforts to overcome stress in quail using VALE also improved egg production and quality [22].

Research of African leaves extract (VALE) in broiler chickens has been widely conducted, while there has been no publication on quail during their laying period related to carcass trait. Therefore, this study aimed to assess the impact of VALE on quail characteristics and carcass quality and observed that appropriate VALE supplementation increased carcass traits and improved final products.

## Materials and Methods

### Ethical approval

The study was approved by the Animal Ethics Committee in the Faculty of Animal Sciences, Diponegoro University, Semarang, Indonesia (Approval number 58-09/A-8/KEP-FPP/2022).

### Study period and location

This study was conducted from September to December 2022 in the Poultry Production Laboratory of the Faculty of Animal and Agricultural Sciences, Diponegoro University.

### *Vernonia amygdalina* leaf extract preparation

This study used African leaves (*V. amygdalina*) that are comprised of young and old leaves bought from a Small Medium Enterprise herb producer (Bibitherbal, Jember City, Indonesia). *Vernonia amygdalina* leaf extract was generated using a previous decoction method [23], where leaves were aerated until dry and then ground. Approximately 50 g *V. amygdalina* powder was boiled for 30 min in 1 L water, left cool for 5 min at room temperature (25°C) and filtered using Whatman No. 41. The filtrate was used for treatments.

### Animals

We used 260 5-week-old quails (average body weight = 129.1 ± 2.2 g) who were raised in an open-sided house. Quails were obtained from Kalisidi Village, West Ungaran District, Semarang Regency, Indonesia. Feed composition: Yellow corn, soybean meal, meat and bone meal, limestone, top mix product from PT Medion (Indonesia), and NaCl. The nutrient ratio was: protein: 21.23%, metabolic energy: 2741.57 kcal/kg/ration, fat: 1.2%, and crude fiber: 2.3%.

### Study design

Our study incorporated a completely randomized design with four treatments and five repetitions (13 quails/repetition) with 4 treatments. *Vernonia amygdalina* leaf extract was added to drinking water, that is, T0: Control, T1: 10 mL/L, T2: 20 mL/L, and T3: 30 mL/L.

### Parameters

Life weight, carcass weight, and meat chemical analyses were performed at the study end (17-week-old), with two quails/experimental units used after 8 h of fasting. Quails were slaughtered according to halal procedures, then scalded, defeathered, and eviscerated [24]. Meat chemical composition analyses were performed on breasts and thighs. Samples were taken separately and stored at −20°C. Meat was removed from bones and homogenized and analyzed for moisture, crude protein, and crude fat content according to Association of Official Agricultural Chemists method [25]. Cholesterol content was determined using an enzymatic colorimetric method; samples were saponified using methanolic potassium hydroxide and Fluitest cholesterol kit (DSI, Indonesia) was used for assay. Results were recorded at 500 nm using a spectrophotometer (Shimadzu, UV1900I, EUROPA GmbH, Germany).

### Statistical analysis

Data were analyzed in a completely randomized design using a one-way analysis of variance and Duncan’s Multiple Range Tests with 95% confidence level (a = 0.05). Analyses were performed using JMP^®^Pro 13.0.0 (64-bit) software (JMP Statistical Discovery LLC, Netherlands). Orthogonal polynomial analyses were performed using MS Excel 16 (Microsoft Corporation, USA).

## Results

### Carcass performance

*Vernonia amygdalina* leaf extract significantly affected carcass weight (p < 0.05), although live weight and carcass and non-carcass percentages were not significantly affected (p > 0.05) (Table-1).

**Table-1:** Carcass performance of Japanese Quail when *Vernonia amygdalina* leaf extract was added to their drinking water.

Parameter	Treatment				p-value

	T0	T1	T2	T3	
Live weight (g)	166.50 ± 4.18	178.50 ± 9.62	174.00 ± 15.47	162 ± 7.37	0.08
Carcass weight (g)	94.45 ± 4.83^b^	103.27 ± 4.23^a^	103.21 ± 9.92^a^	90.84 ± 7.50^b^	<0.01
Percentage of carcass (%)	56.75 ± 3.23	57.93 ± 2.51	59.31 ± 2.08	56 ± 2.10	0.22
Percentage of non-carcass (%)	24.50 ± 1.95	24.79 ± 1.38	25.30 ± 1.64	24.14 ± 1.54	0.73

^a,b^Means with different superscripts along the same row are significantly different (p < 0.01)

### Meat quality

*Vernonia amygdalina* leaf extract (T1, T2, and T3) did not significantly (p > 0.05) affect fat and protein content but significantly (p < 0.05) affected cholesterol levels in meat (Table-2). Carcass cholesterol levels were decreased significantly (p < 0.05) in the T1 group but had decreased more in T2. T3 and T2 groups were not significantly different (p > 0.05).

**Table-2:** The chemical quality of quail meat when *Vernonia amygdalina* leaf extract was added to the drinking water.

Parameter	Treatment				p-value

	T0	T1	T2	T3	
Moisture (%)	73.74 ± 0.45	72.08 ± 0.45	72.23 ± 0.55	72.70 ± 0.84	0.08
Fat (%)	1.57 ± 0.22	1.79 ± 0.24	1.80 ± 0.44	1.84 ± 0.21	0.49
Protein (%)	21.22 ± 0.56	21.91 ± 0.48	21.98 ± 1.24	22.73 ± 0.87	0.22
Cholesterol (mg/100 g)	294.31 ± 47.09^a^	226.12 ± 24.02^b^	167.85 ± 30.1^c^	164.46 ± 41.1^c^	0.01

^a,b,c^Means with different superscript along the same row are significantly different (p < 0.01)

### Carcass physical qualities

*Vernonia amygdalina* leaf extract significantly (p < 0.05) affected water holding capacity (WHC) in breast meat, but did not significantly affect (p > 0.05) pH, drip loss, breast and tight meat color, and WHC of thigh meat (Table-3).

**Table-3:** Physical properties of meat quail when *Vernonia amygdalina* extract leaf extract was added to the drinking water.

Parameter	Treatment				p-value

	T0	T1	T2	T3	
pH	6.36 ± 0.54	6.28 ± 0.39	6.30 ± 0.38	6.26 ± 0.46	0.28
Breast	6.36 ± 0.54	6.28 ± 0.39	6.30 ± 0.38	6.26 ± 0.46	0.28
Thigh	6.78 ± 0.29	6.70 ± 0.24	6.82 ± 0.13	6.82 ± 0.18	0.28
WHC					
Breast	23.76 ± 1.17^b^	24.21 ± 1.70^b^	23.43 ± 0.90^b^	30.81 ± 1.84^a^	<0.01
Thigh	35.15 ± 16.73	41.50 ± 11.98	39.27 ± 9.25	38.31 ± 7.03	0.95
Drip loss					
Breast	5.79 ± 0.84	5.12 ± 0.86	5.98 ± 0.10	5.59 ± 1.30	0.89
Thigh	5.66 ± 1.40	5.36 ± 1.69	4.22 ± 1.99	4.21 ± 1.85	0.95
Color					
L* Breast	26.90 ± 5.66	23.38 ± 8.94	27.70 ± 11.10	27.30 ± 9.65	0.91
L* Thigh	37.60 ± 6.82	37.60 ± 11.54	37.50 ± 14.56	38.00 ± 13.38	0.89
a* Breast (a)	15.50 ± 1.66	14.60 ± 1.47	15.80 ± 1.20	15.40 ± 10.74	0.90
a*Thigh (a)	16.20 ± 1.04	16.60 ± 1.08	16.50 ± 2.52	28.75 ± 6.08	0.68
b* Breast	3.13 ± 0.85	2.60 ± 0.96	3.10 ± 1.56	2.20 ± 0.67	0.51
b* Thigh	4.00 ± 0.91	4.10 ± 1.47	3.90 ± 1.08	4.10 ± 2.79	0.28

L*=Lightness, a*=Redness, b*=Yellowness. ^a,b^Means with different superscripts along the same row are significantly different (p < 0.01), WHC = Water holding capacity

## Discussion

Live weight was not significantly different between groups (p > 0.05), but VALE supplementation significantly increased carcass weight (p < 0.05) (Table-1). This result was similar to Osho *et al*. [26], who showed that VALE (aqueous) supplementation (up to 2.5 g/L) did not significantly affect live weight, while Japhet and Godgift [19] reported that VALE supplementation (50 mL/L) did not significantly affect live pullet weight, but increased live broiler weight. However, Daramola *et al*. [27] and Tokofai *et al*. [28] reported that VALE (100 mg/kg body weight) increased 42-day-old broiler live weight. In our study, live weight was not significantly different between groups because our observations were made during laying periods. After entering this period, quails do not experience many body weight changes. This result concurred with Korwin-Kossakowska *et al*. [29], who reported little change in female quail body weight during laying periods and from the same generation unless nutrients were used.

*Vernonia amygdalina* leaf extract (10 mL/L and 20 mL/L: T1 and T2, respectively) significantly (p < 0.05) increased carcass weight, but this was reduced at 30 mL/L (T3), while carcass and non-carcass percentages were not significantly different (Table-1). These observations concurred with the previous studies: Japhet and Godgift [19] reported that carcass percentages did not always have positive correlation with carcass weights. Daramola *et al*. [27] stated that *V. amygdalina* leaf powder (0.3%) significantly increased carcass weight. Rusli *et al*. [30] recorded that *V. amygdalina* leaf powder (2–6%) did not significantly affect carcass percentages. Mandey *et al*. [20] reported that *V. amygdalina* leaf juice (20 mL/L) did not significantly affect broiler carcass weight, while 30 mL/L supplementation significantly increased carcass weight but not carcass weight percentages. Abdel-Moneim *et al*. [8] stated that heat stress reduced intestinal function and protein digestibility. Ugokwe and Ugokwe [31] reported that VALE significantly increased duodenal, jejunal, ileal villus height, and crypt depth. Thus nutrient absorption and growth indices in broilers were better, while higher doses (up to 100 mg/g) did not induce significant changes. In our study, VALE (10 mL/L and 20 mL/L in T1 and T2 groups, respectively) significantly increased carcass weight (p < 0.05), but 30 mL/L did not (p > 0.05). Thus, low VALE doses improved carcass weights, while 30 mL/L had no significant effects (p > 0.05); this was possibly attributed to reduced nutrient bioavailability from high anti-nutritional substances in extracts. Increased carcass weights without increased non-carcass percentages (Table-1) suggested VALE increased meat production.

Quail carcass moisture and meat protein levels were not significantly different (p > 0.05) (Table-2). *Vernonia amygdalina* leaf extract increased nutrient digestibility and absorption only to increase carcass weight, but was insufficient in increasing carcass meat protein levels. Quails were maintained in open cages at 23.9°C–33.5°C and humidity = 85–90%, so they were potentially stressed. Research data on stress reduction by VALE are often inconsistent. Oyesola *et al*. [14] stated that *V. amygdalina* contained tannin, alkaloid, phenol, and flavonoid antioxidants and were effective against oxidative stress. Antioxidant feed supplementation increased feed nutrient digestibility and absorption [32, 33]. Vargas-Sánchez *et al*. [10] reported that natural herb antioxidant actions mostly improved quail carcass quality without affecting carcass weight. In our study, VALE, as an antioxidant herb source, effectively increased carcass weight. The type, conformance, and concentration of active compounds determined its effects; too high concentrations decreased absorption and metabolism indices [10]. Only one study reported effective antioxidant supplementation on increasing protein deposition in carcasses [18].

Fat content was not significantly different (p > 0.05), but cholesterol levels were significantly decreased (p < 0.05) and were relatively lower when compared with Ilavarasan *et al*. [34], who showed that fat levels were 2.5–2.78%. Omede *et al*. [13], Praptiwi *et al*. [35], and Nowak *et al*. [11] reported that *V. amygdalina* contained high flavonoid levels, which reduced bird meat fat [34, 36–39]. Furthermore, Tan *et al*. [40] showed that flavonoids inhibited adipogenesis and increased lipolysis and apoptosis in adipose tissue cells, with reduced meat fat. In our study, fat levels were not significantly different between groups.

*Vernonia amygdalina* leaf extract significantly (p < 0.05) affected meat cholesterol. At 10 mL/L VALE (T1), cholesterol levels were decreased and were reduced at 20 mL/L (T2), but T2 and T3 groups were not significantly different. *Vernonia amygdalina* leaf extract contains antioxidants that reduce bile acid secretion and cholesterol deposition in meat, in agreement with Suhaemi and Hidayati [21], who showed that *V. amygdalina* leaf powder (1–2%) reduced cholesterol levels in broiler meat. *Vernonia amygdalina* contains many antioxidants [41, 42] which inhibit HMG CoA reductase and lipase enzymes [43]. Reduced lipase activity is followed by reduced cholesterol synthesis [44, 45] and reduced meat cholesterol levels [34]. Vargas-Sánchez *et al*. [10] indicated that particular herb antinutritive and or active compound (e.g., tannin) at high concentrations reduced carcass quality. Cholesterol levels in T2 and T3 groups were non-significantly different and showed that high VALE values did not reduce meat cholesterol levels.

*Vernonia amygdalina* leaf extract at 10–30 mL/L concentrations did not significantly affect (p > 0.05) physical carcass parameters, except WHC (Table-3). We hypothesized that bioactive *Vernonia amygdalina* leaf extract improved carcass production (Table-1), improved chemical (Table-2) and physical meat qualities, especially WHC (Table-3). Improved meat quality is related to increased antioxidant levels and activity [46], thus, our observations may reflect increased antioxidant levels. Victoria *et al*. [46] showed that 7.5% African leaf meal significantly increased physical properties in broiler meat, that is, pH, WHC, and drip loss, but meat color was not significantly changed. These observations concurred with Hosseindoust *et al*. [47], who showed that natural antioxidant addition did not affect other meat quality parameters, including meat color.

To determine optimum VALE doses, we used polynomial orthogonal analysis (Table-4), which showed strong R^2^ values for cholesterol (0.720) and WHC (0.701), but weak values for carcass weight (0.066). Based on the equation that was formed, the optimum VALE dose to produce low meat cholesterol levels was 28.821 mL/L VALE in drinking water that was predicted to reach an optimized minimum cholesterol level o*f* 161.992 g/100 g. Optimization of quail meat WHC values obtained at VALE dose of 9.141 mL/L.

**Table-4:** Parameters, equation, R^2^ value, VALE dosage, and predicted observed parameter optimized value as polynomial orthogonal analysis results.

Parameter	Equation	R^2^	Predicted VALE dosage	Optimized value of the observed parameter
Cholesterol (mg/100 g)	Y = 0.162×^2^ – 9.3381× + 296.56	0.720	28.821	161.992
Carcass weight (g)	Y = −0.003×^2^ + 0.5804× + 95.279	0.066	96.730	123.351
WHC (%)	Y = 0.0191×^2^ – 0.3492× + 23.6	0.701	9.141	22.004

WHC=Water holding capacity, VALE*=Vernonia amygdalina* Del. leaf extract

## Conclusion

*Vernonia amygdalina* leaf extract (20 mL/L) improved quail carcass traits, especially cholesterol levels in quail meat and carcass weight. The optimum efficacy of VALE in reducing quail meat cholesterol might be achieved with a higher dosage, however, it can lower carcass weight.

## Authors’ Contributions

SK and TAS: Prepared the materials, planned and designed the study, recorded and analyzed the data, and drafted and revised the manuscript. LDM and DSP: Prepared the materials, conducted the study, and drafted and revised the manuscript. All authors have read, reviewed, and approved the final manuscript.
